# Sexual Harassment, Abuse, and Discrimination in Obstetrics and Gynecology: A Systematic Review

**DOI:** 10.1001/jamanetworkopen.2024.10706

**Published:** 2024-05-08

**Authors:** Ankita Gupta, Jennifer C. Thompson, Nancy E. Ringel, Shunaha Kim-Fine, Lindsay A. Ferguson, Stephanie V. Blank, Cheryl B. Iglesia, Ethan M. Balk, Angeles Alvarez Secord, Jeffrey F. Hines, Jubilee Brown, Cara L. Grimes

**Affiliations:** 1 Division of Urogynecology, University of Louisville Health, Louisville, Kentucky; 2 Division of Urogynecology, Department of Obstetrics and Gynecology, Northwest Kaiser Permanente, Portland, Oregon; 3 Division of Urogynecology and Reconstructive Pelvic Surgery, Department of Obstetrics, Gynecology, and Reproductive Sciences, Yale University School of Medicine, New Haven, Connecticut; 4 Department of Obstetrics and Gynecology, Cumming School of Medicine, University of Calgary, Calgary, Alberta, Canada; 5 Division of Gynecologic Oncology, University Hospitals Cleveland Medical Center/Case Western Reserve University, Cleveland, Ohio; 6 Division of Gynecologic Oncology, Icahn School of Medicine at Mount Sinai, New York, New York; 7 Division of Urogynecology, MedStar Health, Washington, District of Columbia; 8 Department of Obstetrics and Gynecology, Georgetown University School of Medicine, Washington, District of Columbia; 9 Center for Evidence Synthesis in Health, Brown University School of Public Health, Providence, Rhode Island; 10 Division of Gynecologic Oncology, Duke University Medical Center, Duke Cancer Institute, Durham, North Carolina; 11 University of Connecticut Health Center, Farmington, Connecticut; 12 Atrium Health Levine Cancer, Charlotte, North Carolina; 13 Department of Obstetrics and Gynecology, New York Medical College and Westchester Medical Center, Valhalla, New York

## Abstract

**Importance:**

Unlike other surgical specialties, obstetrics and gynecology (OB-GYN) has been predominantly female for the last decade. The association of this with gender bias and sexual harassment is not known.

**Objective:**

To systematically review the prevalence of sexual harassment, bullying, abuse, and discrimination among OB-GYN clinicians and trainees and interventions aimed at reducing harassment in OB-GYN and other surgical specialties.

**Evidence Review:**

A systematic search of PubMed, Embase, and ClinicalTrials.gov was conducted to identify studies published from inception through June 13, 2023.**:** For the prevalence of harassment, OB-GYN clinicians and trainees on OB-GYN rotations in all subspecialties in the US or Canada were included. Personal experiences of harassment (sexual harassment, bullying, abuse, and discrimination) by other health care personnel, event reporting, burnout and exit from medicine, fear of retaliation, and related outcomes were included. Interventions across all surgical specialties in any country to decrease incidence of harassment were also evaluated. Abstracts and potentially relevant full-text articles were double screened.**:** Eligible studies were extracted into standard forms. Risk of bias and certainty of evidence of included research were assessed. A meta-analysis was not performed owing to heterogeneity of outcomes.

**Findings:**

A total of 10 eligible studies among 5852 participants addressed prevalence and 12 eligible studies among 2906 participants addressed interventions. The prevalence of sexual harassment (range, 250 of 907 physicians [27.6%] to 181 of 255 female gynecologic oncologists [70.9%]), workplace discrimination (range, 142 of 249 gynecologic oncologists [57.0%] to 354 of 527 gynecologic oncologists [67.2%] among women; 138 of 358 gynecologic oncologists among males [38.5%]), and bullying (131 of 248 female gynecologic oncologists [52.8%]) was frequent among OB-GYN respondents. OB-GYN trainees commonly experienced sexual harassment (253 of 366 respondents [69.1%]), which included gender harassment, unwanted sexual attention, and sexual coercion. The proportion of OB-GYN clinicians who reported their sexual harassment to anyone ranged from 21 of 250 AAGL (formerly, the American Association of Gynecologic Laparoscopists) members (8.4%) to 32 of 256 gynecologic oncologists (12.5%) compared with 32.6% of OB-GYN trainees. Mistreatment during their OB-GYN rotation was indicated by 168 of 668 medical students surveyed (25.1%). Perpetrators of harassment included physicians (30.1%), other trainees (13.1%), and operating room staff (7.7%). Various interventions were used and studied, which were associated with improved recognition of bias and reporting (eg, implementation of a video- and discussion-based mistreatment program during a surgery clerkship was associated with a decrease in medical student mistreatment reports from 14 reports in previous year to 9 reports in the first year and 4 in the second year after implementation). However, no significant decrease in the frequency of sexual harassment was found with any intervention.

**Conclusions and Relevance:**

This study found high rates of harassment behaviors within OB-GYN. Interventions to limit these behaviors were not adequately studied, were limited mostly to medical students, and typically did not specifically address sexual or other forms of harassment.

## Key Points

**Question:**

What is the prevalence of sexual harassment, bullying, abuse, workplace discrimination, and other forms of harassment among medical students, residents, fellows, and attending physicians in obstetrics and gynecology (OB-GYN)?

**Findings:**

In this systematic review of 10 studies of harassment among 5852 participants and 12 studies among 2906 participants of interventions, sexual harassment (range, 28%-71%), workplace discrimination (range, 57%-67% among females; 39% among males), and bullying (53%) were frequent among OB-GYN respondents.

**Meaning:**

These findings suggest that there is high prevalence of harassment in OB-GYN despite the field being a female dominant for the last decade.

## Teaser

This systematic review investigates the prevalence of harassment, including sexual harassment, bullying, and abuse, among obstetrics and gynecology medical students and clinicians.

## Introduction

Bullying, sexual harassment, and discrimination are pervasive across society, and mistreatment is often based on personal characteristics or demographics, such as sex, gender, and race and ethnicity. A 2021 systematic review^1^ found that within academic medicine, bullying commonly involved overwork and was associated with negative outcomes for well-being and psychological distress. Academic bulling was associated with 44% of women reporting loss of career opportunities and 32% of men experiencing decreased confidence.^1^ Unlike bullying, which can be more amorphous, sexual harassment in the workplace comprises 3 major forms: sexual coercion, consisting of using professional rewards or threats for sexual favors; unwanted sexual attention, such as unwelcome advances, touching, assault, or rape; and gender harassment, referring to offensive verbal slurs, gestures, or sexist remarks like “Women don’t belong in surgery.”^2,3^ In 2018, the National Academies of Sciences (NAS) found that sexual harassment was highly prevalent, with more than 45% of women in medicine experiencing sexist hostility and 18% experiencing crude behavior. Findings confirmed that sexual harassment is associated with impeded professional and educational goal attainment for women, undermined research integrity, a reduced talent pool, and negative physical and mental health outcomes among targets and bystanders.^3^

Building on the NAS report, several authors reported even higher rates of harassment in women^4^ and extended findings to include men, transgender and gender nonbinary individuals, and those with intersectional identities across various medical subspecialties.^5^ In 2023, harassment in various forms was reported via traditional media outlets and digital and social media. This led to multiple society statements condemning harassment and violence in medicine and a commitment by the American College of Obstetricians and Gynecologists^6^ and other professional societies, including the Society of Gynecologic Surgeons (SGS) and Society of Gynecologic Oncology (SGO), to address needs of professional members.^7^ The joint SGS/SGO statement, endorsed by 11 other societies and foundations, outlines the expectation that members uphold principles of ethical conduct; categorically opposes and condemns sexual or verbal harassment of any kind; reiterates that all people should be treated with dignity, respect, and compassion; and provides resources to individuals experiencing harassment.^7^ The purpose of this systematic review was to investigate the prevalence of sexual harassment, bullying, abuse, workplace discrimination, and other forms of harassment in the obstetrics and gynecology (OB-GYN) field and evaluate interventions to reduce harassment across surgical specialties.

## Methods

This systematic review was conducted as a joint venture between the SGS Systematic Review Group and SGO using standard systematic review methodology, including an a priori protocol (PROSPERO registration, CRD42023439415). The Preferred Reporting Items for Systematic Reviews and Meta-analyses (PRISMA) reporting guideline was followed. The University of Louisville Institutional Review Board determined that this systematic review did not require institutional review board approval because the project did not meet the Common Rule definition of human participant research.

The 12-member working team comprised gynecologists (A.G. and S.K.F.), urogynecologists (A.G., J.C.T., N.E.R., S.K.F., C.B.I., and C.L.G.), gynecologic oncologists (L.A.F., S.V.B., A.A.S., J.F.H., and J.B.), and a systematic review methodologist (E.M.B.). We searched PubMed, Embase, and ClinicalTrials.gov from inception to June 13, 2023. Full search strategies included broad terminology to identify interventions and outcomes of interest in surgical specialties (eAppendix 1 in Supplement 1). Reference lists of similar systematic reviews were also screened.

We evaluated workplace harassment among and by health care workers. We excluded harassment by patients or family members. Eligibility criteria for prevalence and intervention studies, along with further details about methods, are described in eAppendix 2 in Supplement 1. Abstracts were screened in duplicate using Abstrackr software (Brown University Center for Evidence Synthesis in Health).^8^ Potentially relevant full-text articles were rescreened in duplicate. We extracted data in duplicate into SRDRplus.^9^

### Statistical Analysis

Prevalence studies were assessed for clarity, completeness of reporting, representativeness of surveyed participants, response rate, and reliability and validity of the survey instrument. Intervention studies were assessed with the Cochrane risk of bias tool, and selected questions from the Risk of Bias in Nonrandomized Studies of Interventions tool were used as applicable per study.^10,11^ Each study was assigned as good, fair, or poor quality based on likelihood of biases, scientific merit, and completeness of reporting.

## Results

**Figure 1.**
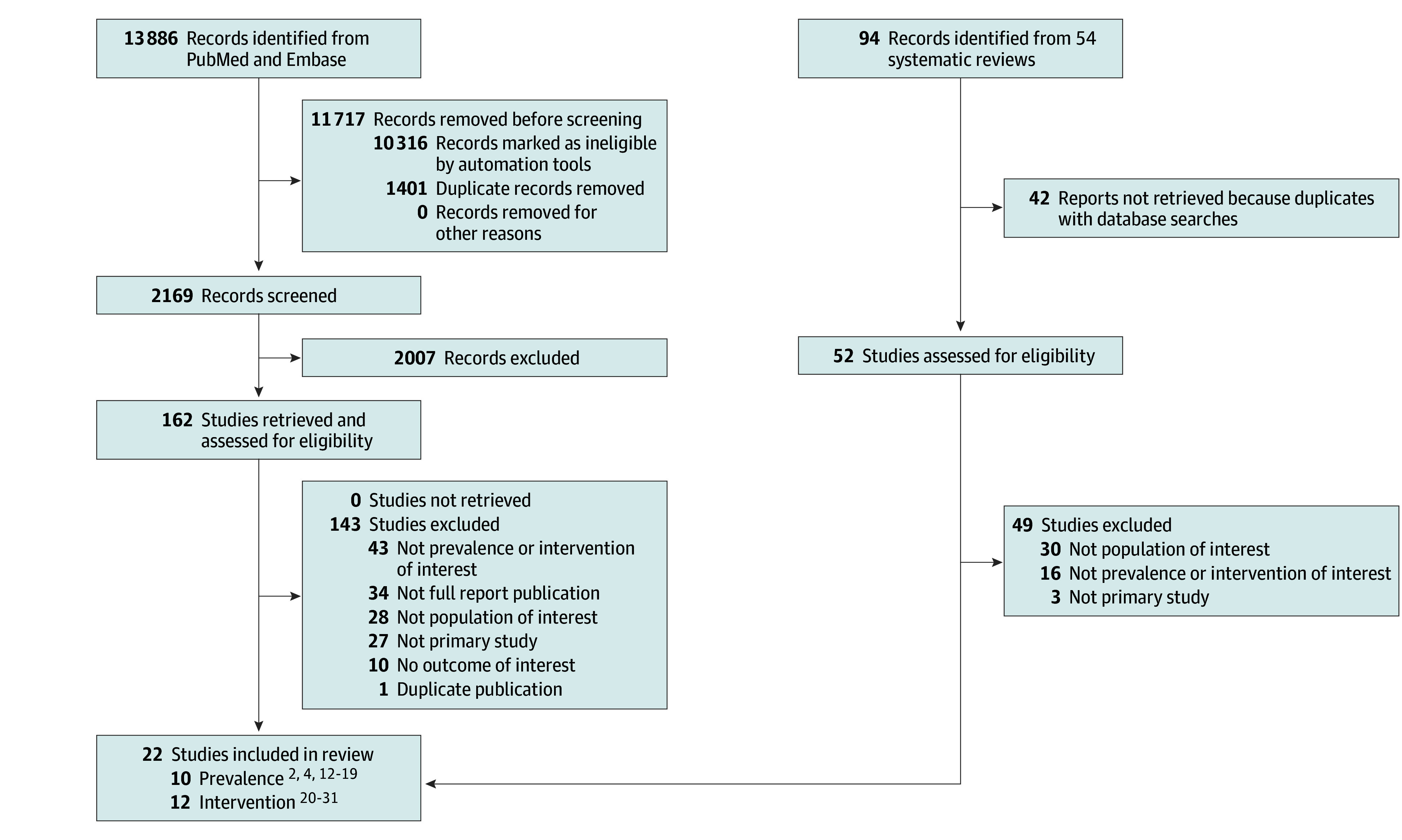
Study Flowchart

The literature search identified 13 886 citations, of which 162 were retrieved for full-text screening. We also screened 54 systematic reviews for relevant references. In total, we included 22 studies that met eligibility criteria; 10 studies among 5852 participants addressed prevalence,^2,4,12,13,14,15,16,17,18,19^ and 12 studies among 2906 participants addressed interventions^20,21,22,23,24,25,26,27,28,29,30,31^ (Figure 1). A meta-analysis was not feasible due to substantial study heterogeneity.

### Prevalence

A total of 10 studies met inclusion criteria for reporting on prevalence of harassment, bullying, and mistreatment in OB-GYN in the US and Canada, including 6 studies^2,4,12,13,15,19^ among 2214 practicing OB-GYN clinicians or OB-GYN clinicians in training and 4 studies^14,16,17,18^ among 3638 medical students surveyed about mistreatment, harassment, belittlement, and verbal and physical abuse while on their OB-GYN clerkship. Studies were predominantly survey based and cross-sectional. Overall, the quality of studies was moderate, with concerns about low response rates (range, 907 of 7026 individuals [12.9%]^13^ to 505 of 513 individuals [98.4%]^16^) (Table 1).

**Table 1.** Studies Reporting on Prevalence

Source	Study design, period	Study quality^a^	Validated survey	Responses, No./total No. (%)	Specialties (% OB-GYN)	Types of harassment
Barnes et al,^12^ 2019	Online survey, NR	A	Yes	33/50 (66.0)	General surgery, neurosurgery, OB-GYN (54.5), orthopedic surgery, ENT	Gender bias, microaggressions
					OB-GYN was considered “female dominant” compared with all other “male-dominant” surgical fields	
Brown et al,^13^ 2019	Survey, 2018	C	No	907/7026 (12.9)	OB-GYN (100)	Workplace discrimination, workplace harassment, nonsexual workplace harassment, sexual harassment
				603/4176 (14.4) US		
				304/2850 (10.7) non-US	AAGL members only	
				175/907 (19.3) Trainees		
Stasenko et al,^4^ 2020	Survey, 2018	C	No	402/1566 (25.7)	Gynecologic oncology (100)	Sexual harassment, gender discrimination
					SGO members only	
Hong et al,^15^ 2022	Survey, 2020	B	Yes	250/452 (55.3)	Gynecologic oncology (100)	Bullying, discrimination, microaggressions
					Women of Gynecologic Oncology only	
Menhaji et al,^2^ 2022	Survey, 2019	B	Yes	404/1473 (27.4)	OB-GYN residents and fellows (100), subspecialty not specified	Sexual harassment: gender harassment, sexist hostility and crude behavior
Sudol et al,^19^ 2021	Survey, 2020	B	Yes	652/1609 (40.5)	General surgery, neurosurgery, OB-GYN (37.1), ophthalmology, orthopedic surgery, otolaryngology, plastic and reconstructive surgery, urology, anesthesia, podiatry	Sexist and racial and ethnic microaggressions
Margitta et al,^17^ 1996	Survey, 1992-1993	B	Yes	301/415 (72.5) response rate over 2 y	Medical students (reporting on all their rotations)	Verbal abuse, physical assault, sexual advances, exposure to pornography
Frank et al,^14^ 2006	Survey at 3 times, 2003	B	No	2316/2884 (80.3)	Medical students (all specialties)	Harassment and belittlement
					91 Respondents who reported on OB-GYN included	
Oser et al,^18^ 2014	Survey, 2003-2010	B	No	801/1059 (75.6)	Medical students (multiple specialties; 668/1059 respondents who reported on OB-GYN included [63.1])	Mistreatment
Kappy et al,^16^ 2019	Retrospective cohort study, 2015-2018	B	No	505/513 (98.4)	OB-GYN clerkship (100)	Students treated in a professional and respectful manner

Abbreviations: AAGL, American Association of Gynecologic Laparoscopy; ENT, ear, nose, and throat; OB-GYN, obstetrics and gynecology; SGO, Society of Gynecologic Oncology.

^a^ Study quality was assigned as good (A), fair (B), or poor (C) based on likelihood of biases, scientific merit, and completeness of reporting.

#### Prevalence of Sexual Harassment

**Figure 2.**
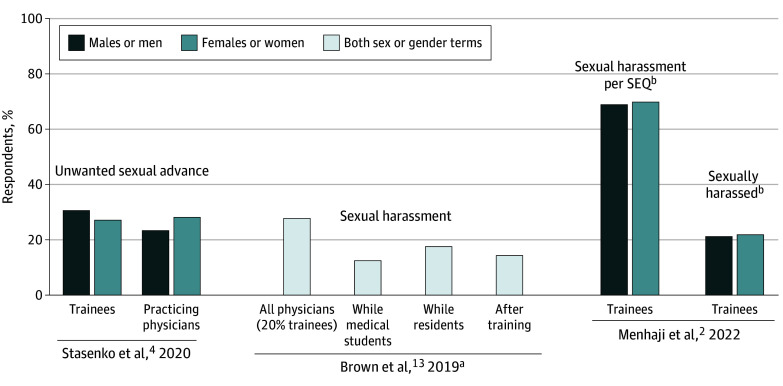
Sexual Harassment in Obstetrics and Gynecology SEQ indicates Sexual Experiences Questionnaire-short form. ^a^Sexual harassment was not reported by sex, but 530 of 907 respondents (58.4%) were female. The percentage of harassment at each training level was calculated based on the assumption that all respondents were medical students or residents prior to independent practice; 26 fellows experienced sexual harassment, but this was not included due to lack of denominator. ^b^Sexual harassment per SEQ was based on any answer other than *never* to any SEQ question. Sexually harassed was based on the answer *yes* to the question “Have you ever been sexually harassed in your training?”

A total of 3 studies queried the prevalence of sexual harassment among OB-GYN clinicians (Figure 2).^2,4,13^ Definitions and reporting of sexual harassment differed by study. A survey of 402 gynecologic oncologists found that 256 respondents (63.6%) had experienced some form of sexual harassment, including unwanted sexual advances, sexist remarks, or the exchanging of sexual favors for an academic position. Sexual harassment was more common among females (181 of 255 respondents [70.9%]) but also commonly occurred among male respondents (75 of 147 respondents [51.0%]).^4^

Among 907 physician members of the AAGL (formerly the American Association of Gynecologic Laparoscopy), 250 respondents (27.6%) reported sexual harassment, including suggestive or offensive stories, attempts to establish a sexual relationship, bribes to engage in sexual behavior, and sexual assault.^13^ Most respondents who had experienced sexual assault were within the US (198 respondents [79.2%]), and 226 perpetrators (90.4%) were physicians.^13^

A survey of 366 OB-GYN trainees found that 253 respondents (69.1%), including 32 of 46 men (69.6%) and 202 of 294 women (68.7%), had experienced sexual harassment based on responses other than *never* on the Sexual Experiences Questionnaire.^2^ This included gender harassment, unwanted sexual attention, and sexual coercion. The largest group of perpetrators consisted of senior OB-GYN attending physicians (30.1%), while 13.1% were residents or fellows, 8.2% were patients, and 7.7% were operating room staff.^2^ While 10.6% of perpetrators were women, they were the perpetrators in 57.7% of cases in which the individual experiencing harassment was a man trainee.^2^

Reporting of sexual harassment to colleagues, supervisors, or other responsible parties varied widely. A total of 32 of 256 gynecologic oncologists (12.5%) and 21 of 250 AAGL members (8.4%) reported their sexual harassment. In the survey of 366 OB-GYN trainees, 32.6% of respondents who experienced harassment reported their harassment, predominantly (71.8%) to another trainee.^2,4,13^ Among respondents who reported their harassment, 8% said that they did not feel that it was taken seriously.^2^ From 63 of 188 individuals (33.5%)^4^ to 80 of 199 individuals (40.2%)^13^ experiencing harassment did not report due to fear of retaliation.

#### Gender Bias and Microaggressions

A total of 5 studies^4,12,13,15,19^ evaluated bias, microaggressions, or workplace discrimination related to gender, sexual orientation, and race among OB-GYN clinicians. One study^12^ considered OB-GYN to be a female-dominant surgical specialty and compared it with other surgical specialties considered to be male dominant (eg, general surgery, orthopedics, neurosurgery, and ear, nose, and throat surgery). Another study^19^ queried multiple surgical specialists, including OB-GYN clinicians, about microaggressions against surgeons based on gender, race, and ethnicity. The other 3 studies^4,13,15^ focused on OB-GYN clinicians.

A survey of 250 female gynecologic oncologists found that 131 of 248 respondents with data (52.8%) reported bullying and 142 of 249 respondents with data (57.0%) reported gender discrimination. Most respondents (208 individuals [83.2%]) reported microaggressions, including being told to smile more, dress in certain ways, or to “act more female” or “motherly.”^15^ In another study of gynecologic oncologists,^4^ 71 of 215 women (33.0%) reported being denied opportunities for training or rewards based on gender compared with 25 of 131 men (19.1%). Although men experienced significantly less workplace discrimination than women (138 of 358 men [38.5%]) vs 354 of 527 women [67.2%]), gender discrimination was the most common form of discrimination for men (99 of 137 men [72.3%]) and women (318 of 353 women [90.1%]) in gynecologic surgery.^13^ Although OB-GYN is considered a female-dominant specialty, among 18 OB-GYN trainees, 17 respondents (94.4%) had been mistaken as nonphysicians, while 16 respondents (88.9%) preapologized for asking for something from a surgical technician or a nurse and 15 respondents (83.3%) needed to make such requests multiple times.^12^ Surgical technicians and circulating nurses were predominantly responsible for these microaggressions (13 respondents [72.2%]).^12^ In another survey-based study assessing burnout as a sequela of microaggressions,^19^ 115 of 218 OB-GYN clinicians reported burnout experiences (52.8%).

#### Medical Student Mistreatment

There were 4 studies^14,16,17,18^ that evaluated medical student experiences on clinical clerkships in OB-GYN. Among 668 medical students in 1 study,^18^ 168 respondents (25.1%) reported occasional or frequent mistreatment (eg, verbal abuse, coercion, or negative consequences) during OB-GYN rotations; resident physicians were the most common source. In another survey-based study^14^ among 91 medical students, almost three-quarters of respondents (65 respondents [71.4%]) reported belittlement and 22 respondents (24.2%) reported harassment by OB-GYN residents. Across all clerkship rotations, including general surgery, OB-GYN was noted to have the lowest professionalism scores.^16^ In a small study from 1992,^17^ 4 of 16 medical students (25.0%) reported that they had experienced physical abuse while on OB-GYN.

### Interventions

Among 12 studies that evaluated interventions, 4 studies^20,21,24,27^ evaluated interventions at the resident level in 258 participants, 1 study^23^ evaluated a facultywide cultural competency program in 148 participants, and 7 studies^22,25,26,28,29,30,31^ evaluated interventions to decrease medical student mistreatment in 2500 participants. Intervention studies included 1 randomized clinical trial,^28^ 6 nonrandomized prospective studies,^21,23,24,25,30,31^ 3 studies with a prospective and retrospective component,^20,27,29^ and 2 studies evaluating a single intervention without comparison.^22,26^ The overall quality of evidence was low owing to incomplete description of intervention or measurement tools and high risk of bias (10 studies [83.3%] rated as poor quality) (Table 2).

**Table 2.** Studies Reporting on Interventions

Source	Study design, period	Study quality^a^	Participants, No./Total No.	Specialties (% OB-GYN)	Type of intervention	Outcome
Botros Brey et al,^20^ 2022	Retrospective and prospective, 2019-2021	C	Short term: 24/26	OB-GYN (11/26 [42.3])	Forum theater	Increased confidence to intervene on behalf of themselves and others
			Long term: 25/26	Urology		
Castillo-Angeles et al,^21^ 2019	Prospective, 2016-2017	C	58 at 1 mo	General surgery (0)	Video-based modules; mixed methods analysis	Resident knowledge and attitudes about abuse
			43/58 at 6 mo			
Korndorffer et al,^23^ 2021	Prospective, 2020	C	Faculty: 77/145 (53.1%)	General surgery (0)	Cultural competency curriculum	Bias on the basis of race, gender, and sexual orientation in the last 12 mo; improved ability to analyze and respond to own and workplace bias
			Staff: 48/135 (35.6%)			
			Residents: 23/103 (22.3%)			
Kulaylat et al,^24^ 2017	Prospective, NR	C	Primary care 62/142 (43.7%)	Primary care, surgery, other (NR)	Paper-based clinical vignettes	Perceptions of potential mistreatment among incoming interns
			Surgery 37/142 (26.1%)		Presented to resident during orientation and divided in small groups for discussion	
			Other specialty 43/142 (30.3%)			
Patnaik et al,^27^ 2023	Retrospective and prospective, 2020-2022	C	32/66 (48.5%) participated and 28/32 (87.5%) completed survey	General surgery residency (0)	Forum theater	Understanding mistreatment; recognizing, intervening, and responding to mistreatment
			At 6 mo, 15/28 (53.6%) responded to survey			
Thivierge et al,^28^ 2020	Randomized clinical trial, 2017	A	129/147; 119 completed and analyzed (80.8%)	Surgery clerkship (0)	Simulation of an intimidation scenario (blinded); other group watched videos (not blinded); control group did not receive training about intimidation	Reporting intimidation after surgical rotation vs other rotations
			Simulation: 40/119 (33.6%)			
			Video: 37/119 (31.1%)			
			Control: 42/119 (35.5%)			
Williams-Karnesky et al,^30^ 2020	Prospective, 2018-2019	B	53/NR (51%) participated in intervention; 76 (83.5%) returned postintervention surveys	Surgical clerkship (0)	Standardized video-based curriculum	Experience and witnessing mistreatment
Fried et al,^22^ 2012	Prospective annual survey, 1996-2008	C	1946/2151 (90.5%)	Medical students at end of third year clerkship (NR)	Gender and Power Abuse Committee	Mistreatment and sexual harassment reporting
Lind et al,^26^ 2020	Prospective annual survey, 2013-2019	C	78%-87% response rate	Medical students (NR)	Ending Mistreatment Task Force	Report of mistreatment by medical students
York et al,^31^ 2021	Prospective, 2019	C	Preintervention survey: 75/102 (73.5%)	Medical Students (NR)	Case-based workshop	Confidence in addressing personally experienced and witnessed bias and microaggression
			Postintervention 83/102 (81.4%)			
			1 mo: 20/102 (19.6%)			
			3 mo: 27/102 (26.5%)			
			8 mo: 31/62, 50.0%			
Lau et al,^25^ 2017	Prospective, 2013-2015 (intervention in 2014)	C	141/164 (85.9%)	Medical Students (NR)	Video and discussion–based mistreatment program	Mistreatments reported
Wagner et al,^29^ 2015	Prospective with historic control, 2013	C	Control: 36/88 (40.9%)	Medical Students (NR)	Reporting module	Intimidation score, satisfaction with anonymity of reports, and value of reports higher; mistreatment reporting by medical students
			Initial survey: 103/122 (84.4%)			
			End of clerkship survey: 157/187 (83.9%)			

Abbreviations: NR, not reported; OB-GYN: obstetrics and gynecology.

^a^ Study quality was assigned as good (A), fair (B), or poor (C) based on likelihood of biases, scientific merit, and completeness of reporting.

#### Institutionwide Interventions

There were 2 studies that described institutionwide initiatives to decrease medical student mistreatment.^22,26^ The Gender and Power Abuse Committee^22^ and the Ending Mistreatment Task Force^26^ created multipronged interventions with support from faculty, administrators, and medical student representatives engaging with hospital medical staff leaders, student body representatives, clinical clerkship directors, and faculty governance leaders. Interventions included a no abuse policy, an ombuds office, improved reporting with prompt action, workshops for medical students and residents, and faculty grand rounds sessions. These interventions were associated with a decrease in reported medical student mistreatment ranging from 62.9% of respondents to 40.3% of respondents over 6 years in 1 study,^26^ although the incidence of sexual harassment remained unchanged at 13.4% (260 of 1940 students) across all 4 study periods in the other study (Table 2).^22^

#### Forum Theater, Video Modules, and Paper-Based Clinical Vignettes

We assessed 4 studies that evaluated video-based discussions,^21,25,28,30^ 1 study that evaluated videos in a multipronged intervention,^26^ 2 studies that evaluated clinical scenarios and case-based workshops to prompt discussion,^24,31^ and 2 studies (by the same author evaluating different medical specialties) that evaluated a forum theater intervention.^20,27^ Forum theater is a learning modality in which learners become participants who watch, respond to, and step in to the play to act out potential solutions while a facilitator debriefs and reinforces key messages.^20^ Target audiences were residents,^20,21,24,27^ medical students,^25,28,30,31^ or faculty^26^ in the specialties of general surgery,^21,24,25,27,28,30^ OB-GYN and urology,^20^ or the entire medical school or class.^26,31^ Overall, programs helped trainees to recognize mistreatment and were associated with improved confidence in intervening on their own behalf or on the behalf of others (Table 2).

#### Addition of Reporting Modules and Other Interventions

There were 3 studies that described programs to improve trainee education regarding mistreatment reporting,^22,25,26^ and 1 study evaluated a real-time, web-based reporting module for medical students on the surgical clerkship.^29^ While students perceived less intimidation and greater satisfaction with systems designed to improve reporting, the decrease in perceived abuse was not statistically significant. The implementation of a video- and discussion-based mistreatment program during a surgery clerkship was associated with a decrease in medical student mistreatment reports from 14 reports the year prior to the mistreatment program to 9 reports in the first year and 4 in the second year after implementation.^25^ Using a real time, web-based reporting module, students with access to modules were less intimidated than students in a control group based on a 1 to 10 intimidation score (4.02 vs 5.31) and faculty (5.26 vs 6.28).^29^

There was 1 study that evaluated a 9-week, departmentwide cultural competency curriculum on bias based on race, ethnicity, sexual orientation, or gender in the surgery department.^23^ The curriculum included formal presentations, role play–based simulation, and small group interactions and engaged faculty, residents, and staff. Among 148 participants, 73.7% reported that these interventions helped to analyze their own bias, 65.5% reported improvement in responding to their own bias, and 68.1% reported an improved ability to respond when they see bias in the workplace.^23^

## Discussion

This systematic review found high rates of sexual harassment, gender bias, bullying, and discrimination within OB-GYN. However, interventions to limit these behaviors have not been adequately studied, were limited to medical students, or did not specifically address sexual or other forms of harassment.

The current literature reports a high prevalence of harassment behaviors directed toward surgical trainees. This was consistent with a systematic review addressing academic bullying that found that 32% of general surgery, 25% of OB-GYN, and 21% of medicine interns and medical students reported bullying.^1^ Another systematic review found that 27% of surgical trainees (including OB-GYN) reported sexual harassment^32^ and a study reviewing harassment rates across multiple medical specialties found that OB-GYN was second only to general surgery as the specialty associated with the highest rates of sexual harassment.^33^ Undermining and bullying behaviors are commonplace in surgical specialties, with several physicians condoning tantrums, swearing, humiliation, and undermining of trainees as a “rite of passage.”^34^ This can create a cycle of mistreatment, as seen when medical students experience high rates of belittlement and harassment from OB-GYN residents, who may be modeling behavior seen in senior physicians.^14,34^ Surgical specialties, including OB-GYN, are also high-pressure environments; combined with perfectionist characteristics seen in surgeons, this can create an environment of bullying and harassment.^34^ Equipping OB-GYN clinicians to be better surgical educators, providing clinical support, and modeling positive behavior may help disrupt the culture of harassment.^34,35^

The power differential between medical trainees and other health care professionals, including physicians and nursing staff, can also lead to underreported abuses of professional power. The role of gender is critical to understanding sexual harassment. Although sexual harassment and gender bias were more commonly reported by female OB-GYN respondents, male OB-GYN respondents also reported high rates of sexual harassment and gender discrimination, often by female perpetrators. This suggests that focus should be on perpetrators and leadership demographics to identify harassment behaviors. Unlike many other surgical specialties, OB-GYN has had an increase in the number of women clinicians, from 47% in 2010 to the majority (61%) in 2021.^36^ Despite high numbers of women OB-GYN residents and overrepresentation of women in residency program director roles, women continue to be underrepresented in departmental leadership, making up 24% of chairs in 2013 and 34% of chairs in 2021.^36,37^ However, the continued high rates of harassment in OB-GYN suggest that simply increasing the number of women in medicine is inadequate to address gender bias and discrimination. Rather, the role of power dynamics should be better studied and addressed to reduce harassment.

The high prevalence of sexual harassment in this review may be due in part to varied definitions of sexual harassment across studies. Sexual harassment can include a broad range of behaviors that humiliate, diminish, and demean a person on the basis of sex or gender, including gender harassment, unwanted sexual attention, and sexual coercion.^2,3^ Most women do not consider or report gender harassment as sexual harassment,^2^ explaining the wide range of reported prevalence depending on terminology used in surveys. Additionally, many women underreport incidents of harassment and sexual assault,^3^ and unclear definitions make it difficult for individuals who have experienced harassment to definitively come forward. All trainees should be better versed in all aspects of harassment to improve recognition and reporting in a confidential way free of fear of retaliation.

Interventions to address these pervasive behaviors would seem to be the obvious next step, but unfortunately, interventions to decrease harassment and specifically sexual harassment have been poorly studied. Successful interventions involved change at an institutional level and required support from multiple levels, including hospital administration, management, and leadership.^26^ While providing tools to educate health care staff about harassment may be associated with improved trainee and bystander confidence in standing up for individuals experiencing harassment, the need to maintain confidentiality in reporting presents an additional challenge.^1,20^ This is especially true in cases of sexual harassment where details may be known only to the perpetrator and the individual experiencing harassment. When physicians are required to report their grievances to immediate supervisors, they may perceive senior physicians as untouchable.^1,38,39^ One viable approach appears to be establishing an office of gender equity, as reported by the Medical University of South Carolina,^38^ comprising university faculty with experience in responding to sexual harassment and interpersonal violence. All complaints are evaluated by an intermediary third party who interviews the accuser and accused separately before coming to a determination, thus protecting the individual reporting harassment and alleged perpetrator.^38^ Additional approaches include the Office of Professionalism developed by the University of Colorado School of Medicine, which provides nonpunitive feedback and makes professionalism a component of promotion.^26^

### Strengths and Limitations

This study has several limitations, with the major limitations related to the heterogenous evidence base, including wide variability of assessed forms of harassment and inconsistent or incompletely defined terminology. Additionally, variations in study participant specialties and subspecialties and level of training precluded meta-analyses across studies. Studies were predominantly survey based and retrospective, with moderate to low quality of evidence. Nonresponse and recall bias may have played a large role given that individuals who have been sexually harassed are less inclined to respond to this type of survey.^2^ Therefore, the prevalence of sexual harassment may be different than that reported here. With 1 exception,^20,27^ each intervention was evaluated by 1 study.

This study also has several strengths. It was a joint collaboration among experienced gynecologists, urogynecologists, and gynecologic oncologists and was conducted using a robust methodology. While other systematic reviews have addressed these topics in general surgery, this study specifically identified studies that included or were limited to OB-GYN to provide data within a surgical specialty that currently is majority female.

## Conclusions

This systematic review found that 28% to 71% of participants reported sexual harassment, sexual coercion, or unwanted sexual advances within the field of OB-GYN in surveys. These events were often not reported to institutional leadership, however, given that individuals experiencing these forms of mistreatment feared retaliation and did not feel that their experiences would be taken seriously. There were also high rates of bullying, gender bias, and microaggressions among trainees and practicing physicians. Interventions to decrease harassment had not been adequately studied, but institutionwide, multipronged approaches with support from varying levels of stakeholders appeared to have the highest efficacy for reductions in mistreatment in medical training. Nevertheless, most interventions were not associated with reduced sexual harassment. National medical and hospital associations and departmental and institutional leaders should use these findings to acknowledge the prevalence of bullying, abuse, and sexual harassment and begin to work collectively on best practices to prevent harassment and discrimination, improve reporting, and intervene once reports of alleged misconduct, abuse, and sexual harassment are received. Future studies should focus on such interventions to improve the practicing climate, model professional behavior, and intervene appropriately when harassment behavior is identified within OB-GYN and medicine at large.

## Notes

Supplement 1. **eAppendix 1.** Search Strategy **eAppendix 2.** Study Methodology

Supplement 2.
Data Sharing Statement
