# Supplementary material for: Sexual Harassment, Abuse, and Discrimination in Obstetrics and Gynecology: A Systematic Review
Source: JAMA Netw Open. 2024 May 8;7(5):e2410706. doi: 10.1001/jamanetworkopen.2024.10706 (PMC11079690; doi:10.1001/jamanetworkopen.2024.10706)
Supplement: Supplement 1. — eAppendix 1. Search Strategy eAppendix 2. Study Methodology [file jamanetwopen-e2410706-s001.pdf]

Supplemental Online Content

Gupta A, Thompson JC, Ringel NE, et al. Sexual harassment, abuse, and discrimination in obstetrics and gynecology: a systematic review. *JAMA Netw Open*. 2024;7(5):e2410706. doi:10.1001/jamanetworkopen.2024.10706

eAppendix 1. Search Strategy
eAppendix 2. Study Methodology

This supplemental material has been provided by the authors to give readers additional information about their work.

© 2024 Gupta A et al. *JAMA Network Open.*

PubMed

("Occupational Stress"[Mesh] OR "Bullying"[Mesh] OR "Sexual Harassment"[Mesh] OR "Harassment, Non-Sexual"[Mesh] OR "Emotional Abuse"[Mesh] OR "Physical Abuse"[Mesh] OR "Sex Offenses"[Mesh] OR "Social Discrimination"[Mesh] OR "Perceived Discrimination"[Mesh] OR "Sexism"[Mesh] OR "Ageism"[Mesh] OR "Racism"[Mesh] OR "Bias, Implicit"[Mesh] OR "Homophobia"[Mesh] OR "Coercion"[Mesh] OR "Workplace Violence"[Mesh] OR "Gender-Based Violence"[Mesh] OR "Rape"[Mesh] OR bully OR bullying OR abuse OR abusive OR harass* OR sexis* OR racis* OR coerc* OR rape OR retaliat* OR intimidat* OR mistreat*)

AND

("Obstetrics and Gynecology Department, Hospital"[Mesh] OR "Gynecology"[Mesh] OR "Obstetrics"[Mesh] OR "Gynecologists"[Mesh] OR "Obstetricians"[Mesh] OR obstetric* OR gynecolog* OR female pelvic medicine OR FPMRS OR maternal-fetal medicine OR MFM OR urogynecolog* OR "Surgical Oncology"[Mesh] OR "Urogenital Neoplasms/surgery"[Mesh] OR (gynecolog* AND oncolog* AND surg*) OR "Urology"[Mesh] OR urolog* OR "General Surgery"[Mesh] OR "Orthopedics"[Mesh] OR "Otolaryngology"[Mesh] OR "Surgery, Plastic"[Mesh] OR (("Internship and Residency"[Mesh] OR "Clinical Clerkship"[Mesh] OR "Education, Medical, Undergraduate"[Mesh] OR resident OR residenc* OR intern OR training OR trainee OR student) AND (surgery OR surgical)))

AND

(("Surveys and Questionnaires"[Mesh] OR survey OR questionnaire OR Mixed-Methods OR "mixed methods" OR "Random Allocation"[Mesh] OR "Clinical Trial" [Publication Type] OR "Double-Blind Method"[Mesh] OR "Single-Blind Method"[Mesh] OR random* OR "Placebos"[Mesh] OR placebo OR ((clinical OR controlled) AND trial*) OR ((singl* OR doubl* OR trebl* OR tripl*) AND (blind* OR mask*)) OR rct OR cross-sectional OR crossover OR cross-over OR cross-over OR "treatment switching" OR "Treatment Switching"[Mesh] OR RCT OR "Randomized Controlled Trial" [Publication Type] OR "Cohort Studies"[Mesh] OR cohort OR "Clinical Trial" [Publication Type] OR "Clinical Trials as Topic"[Mesh] OR follow-up OR followup OR longitudinal OR "Placebos"[Mesh] OR placebo* OR "Research Design"[Mesh] OR "Evaluation Studies" [Publication Type] OR "Evaluation Studies as Topic"[Mesh] OR "Comparative Study" [Publication Type] OR ((comparative OR Intervention) AND study) OR pretest* OR pre test* OR posttest* OR post test* OR prepost* OR pre post* OR "before and after" OR interrupted time* OR time serie* OR intervention* OR ((quasi-experiment* OR quasiexperiment* OR quasi experiment*) AND (method OR study OR trial OR design*)) OR "Case-Control Studies"[Mesh] OR (case AND control) OR "Clinical Study" [Publication Type] OR "Clinical Studies as Topic"[Mesh] OR Observational Study [Publication Type] OR "Epidemiologic Studies"[Mesh] OR "Case-Control Studies"[Mesh] OR "Cohort Studies"[Mesh] OR "Case control" OR cohort OR (observational AND (study OR studies)) OR Longitudinal OR Retrospective OR "Prospective Studies"[Mesh] OR "Longitudinal Studies"[Mesh] OR "Follow-Up Studies"[Mesh] OR ((follow-up OR followup OR "follow up") AND (study OR studies)) OR "Registries"[Mesh] OR register[tiab] OR registry[tiab] OR database[tiab] OR "Evaluation Study" [Publication Type] OR "Evaluation Studies as Topic"[Mesh] OR "Validation Study" [Publication Type] OR "Validation Studies as Topic"[Mesh] OR systematic[sb] OR "Meta-Analysis" [Publication Type] OR "Meta-Analysis as Topic"[Mesh] OR "Systematic Review" [Publication Type] OR "Systematic Reviews as Topic"[Mesh] OR "Review Literature as Topic"[Mesh] OR medline[tiab] OR pubmed[tiab] OR cochrane[tiab] OR embase[tiab] OR psychlit[tiab] OR psyclit[tiab] OR psychinfo[tiab] OR psycinfo[tiab] OR cinahl[tiab] OR cinhal[tiab] OR science citation index[tiab] OR bids[tiab] OR cancerlit[tiab] OR reference list*[tiab] OR bibliograph*[tiab] OR hand-search*[tiab] OR relevant journals[tiab] OR manual search*[tiab] OR selection criteria[tiab] OR data extraction[tiab]) NOT ("Address" [Publication Type] OR "autobiography"

© 2024 Gupta A et al. *JAMA Network Open.*

[publication type] OR "bibliography" [publication type] OR "biography" [publication type] OR "case reports" [publication type] OR "comment" [publication type] OR "congress" [publication type] OR "Congresses as Topic"[Mesh] OR "dictionary" [publication type] OR "directory" [publication type] OR "editorial" [publication type] OR "festschrift" [publication type] OR "Government Publication" [Publication Type] OR "Government Publications as Topic"[Mesh] OR "historical article" [publication type] OR "interview" [publication type] OR "lecture" [publication type] OR "legal case" [publication type] OR "legislation" [publication type] OR "letter" [publication type] OR "news" [publication type] OR "newspaper article" [publication type] OR "patient education handout" [publication type] OR "periodical index" [publication type] OR "comment on" OR ("Animals"[Mesh] NOT "Humans"[Mesh]) OR rats[tw] OR cow[tw] OR cows[tw] OR chicken*[tw] OR horse[tw] OR horses[tw] OR mice[tw] OR mouse[tw] OR bovine[tw] OR sheep OR ovine OR murinae))

© 2024 Gupta A et al. *JAMA Network Open.*

Embase

No.	Query
#31.	#30 AND ('case control study'/de OR 'clinical article'/de OR 'clinical study'/de OR 'clinical trial'/de OR 'cohort analysis'/de OR 'comparative study'/de OR 'controlled clinical trial'/de OR 'controlled study'/de OR 'cross sectional study'/de OR 'human'/de OR 'human experiment'/de OR 'interview'/de OR 'longitudinal study'/de OR 'major clinical study'/de OR 'meta analysis'/de OR 'multicenter study'/de OR 'observational study'/de OR 'prospective study'/de OR 'qualitative research'/de OR 'quality control'/de OR 'questionnaire'/de OR 'randomized controlled trial'/de OR 'retrospective study'/de OR 'secondary analysis'/de OR 'semi structured interview'/de OR 'structured interview'/de OR 'structured questionnaire'/de OR 'systematic review'/de OR 'total quality management'/de)
#30.	#17 AND #29
#29.	#18 OR #19 OR #20 OR #21 OR #22 OR #23 OR #24 OR #25 OR #26 OR #27 OR #28
#28.	('student' OR 'medical student' OR trainee OR 'resident' OR 'residency education' OR 'medical education' OR 'fellowship' OR 'internship') AND ('surgeon' OR 'surgery')
#27.	'plastic surgery'
#26.	'otorhinolaryngology'
#25.	'orthopedics'
#24.	'general surgery'
#23.	'urology'
#22.	'gynecologic oncology'
#21.	'maternal fetal medicine'
#20.	'urogynecology' OR 'urogynecologist'
#19.	'obstetrician' OR 'obstetrics'
#18.	'gynecology' OR 'gynecologist'
#17.	#1 OR #2 OR #3 OR #4 OR #5 OR #6 OR #7 OR #8 OR #9 OR #10 OR #11 OR #12 OR #13 OR #14 OR #15 OR #16
#16.	'harassment'
#15.	mistreatment
#14.	'intimidation'
#13.	retaliation
#12.	'rape'
#11.	'gender based violence'
#10.	'coercion'
#9.	'homophobia'
#8.	'racism'
#7.	'sexism' OR 'gender bias'
#6.	'social discrimination'
#5.	'sexual crime'
#4.	'physical abuse'
#3.	'emotional abuse'
#2.	'sexual harassment'
#1.	'bullying'/exp OR 'bullying' OR 'bullying victimization'/exp OR 'bullying victimization'

© 2024 Gupta A et al. *JAMA Network Open.*

Appendix 2: Study methodology

For prevalence, we included physicians and medical students who have completed or are engaged in OBGYN training in the US or Canada. This included medical students who rotated through OBGYN, current residents, and fellows. We evaluated rates of personal experiences of sexual harassment, bullying, abuse and discrimination. Discrimination could be based on race/ethnicity, gender, or sexual orientation. We did not limit to specific definitions of these terms and studies defined these terms in varying ways. When data on OBGYNs were grouped with other specialties but not reported by specialty, we included only studies in which OBGYNs comprised at least 20% of the sample. We also explored victim demographics, perpetrator demographics, rate of event reporting, outcome of event reporting, sequelae of event, fear of retaliation, and demographics of leadership.

To evaluate interventions to decrease bias and harassment, we evaluated all surgical specialties, at any level of training or practice (including medical students), in any country. Few studies reported on interventions within OBGYN and therefore study inclusion was expanded to interventions in all surgical specialties to report on effective interventions that can be applied to OBGYN. The interventions were required to study longitudinal effects in reducing the incidence of bullying, harassment, sexual harassment or trainee mistreatment. We did not further restrict by study design or size. Studies were limited to human subjects without language restriction. We excluded conference presentations. Authors were not contacted for additional information.

Abstracts were screened in duplicate in the validated machine learning software Abstrackr (http://abstrackr.cebm.brown.edu, Brown University, Providence, RI) with reconciliation conducted in conference with all team members or by the first author (AG). Potentially relevant full-text articles were screened by one reviewer and rescreened by a second team member; conflicts were adjudicated by the first author (AG). We extracted data on study design, baseline characteristics, interventions, outcomes, and results into SRDRplus (https://srdrplus.ahrq.gov, AHRQ, Rockville, MD) in separate forms for the questions of prevalence and intervetions, which can be viewed at https://srdrplus.ahrq.gov/projects/4408

© 2024 Gupta A et al. *JAMA Network Open.*

and https://srdrplus.ahrq.gov/projects/4416 . Each accepted study was extracted by one researcher and confirmed by a second. We extracted data in duplicate in to SRDRplus (https://srdrplus.ahrq.gov, AHRQ, Rockville, MD), which can be viewed at https://srdrplus.ahrq.gov/projects/4408 and https://srdrplus.ahrq.gov/projects/4416 .

Prevalence studies were assessed for representativeness of the sample, survey response rate and missing data, reported reliability and validity of the survey instrument, and other biases. All studies were assessed for clarity and completeness of reporting. Intervention studies were assessed with the Cochrane Risk of Bias tool and selected questions from the Risk of Bias in Nonrandomized Studies of Interventions tool related to comparability of intervention groups and adjustment for confounders. Each study was thus assigned as good (A), fair (B), or poor (C) based on likelihood of biases, scientific merit, and completeness of reporting. Studies were summarized narratively and in tables describing their characteristics, findings, and risks of bias.

© 2024 Gupta A et al. *JAMA Network Open.*
