# Supplementary material for: Sexual Harassment, Abuse, and Discrimination in Obstetrics and Gynecology: A Systematic Review
Source: JAMA Netw Open. 2024 May 8;7(5):e2410706. doi: 10.1001/jamanetworkopen.2024.10706 (PMC11079690; doi:10.1001/jamanetworkopen.2024.10706)
Supplement: Supplement 2. — Data Sharing Statement [file jamanetwopen-e2410706-s002.pdf]

Data Sharing Statement

Gupta A. Sexual Harassment, Abuse, and Discrimination in Obstetrics and Gynecology: A Systematic Review. *JAMA Netw Open*. Published online May 8, 2024. doi:10.1001/jamanetworkopen.2024.10706

Data
Data available: Yes
Data types: Data (not involving human participants)
How to access data: https://srdrplus.ahrq.gov/projects/4408 https://srdrplus.ahrq.gov/projects/4416
When available: With publication

Supporting Documents
Document types: Statistical/analytic code
How to access documents: Appendix 1
When available: With publication

Additional Information
Who can access the data: anyone requesting the data **Types of analyses:** any purpose
Mechanisms of data availability: with investigator support
